# Supplementary material for: Structural snapshots of Pseudomonas aeruginosa LptB2FG and LptB2FGC reveal insights into lipopolysaccharide recognition and transport
Source: Nat Commun. 2025 Dec 17;16:11384. doi: 10.1038/s41467-025-66182-0 (PMC12738788; doi:10.1038/s41467-025-66182-0)

Structural snapshots of *Pseudomonas aeruginosa* $LptB_2FG$ and $LptB_2FGC$ reveal insights into lipopolysaccharide recognition and transport

Francesco Fiorentino[1,2], Matteo Cervoni[3], Yi Wang[4], Leonhard H. Urner[5], Joshua B. Sauer[6], Anh Tran[7], Robin A Corey[8], Dante Rotili[3], Antonello Mai[9], Phillip J Stansfeld[10], Francesco Imperi[3], Edward W. Yu[7], Chih-Chia Su[7,*], Carol V. Robinson[2,11,*], Jani R Bolla[4,11,*]

[1]*Department of Biochemical Sciences, Sapienza University of Rome, Piazzale Aldo 5, 00185, Rome, Italy.*

[2]*Department of Chemistry, University of Oxford, South Parks Road, Oxford, OX1 3QZ, UK.*

[3]*Department of Science, Roma Tre University, Viale Marconi 446, 00146, Rome, Italy.*

[4]*Department of Biology, University of Oxford, South Parks Road, Oxford, OX1 3RB, UK.*

[5]*Department of Chemistry and Chemical Biology, TU Dortmund University, Otto-Hahn-Str. 6, 44227 Dortmund, Germany.*

[6]*Department of Biochemistry, University of Oxford, Oxford, South Parks Road, Oxford, OX1 3QU, UK.*

[7]*Department of Pharmacology, Case Western Reserve University School of Medicine, Cleveland, OH 44106, USA.*

[8]*School of Physiology, Pharmacology & Neuroscience, University Walk, BS8 1TD, Bristol, UK.*

[9] *Department of Drug Chemistry and Technologies, Sapienza University of Rome, Piazzale Aldo 5, 00185, Rome, Italy.*

[10]*School of Life Sciences, Gibbet Hill Campus, The University of Warwick, Coventry, CV4 7AL, UK.*

[11]*Kavli Institute for Nanoscience Discovery, University of Oxford, Dorothy Crowfoot Hodgkin Building, University of Oxford, South Parks Road, Oxford, OX1 3QU, UK.*

[*]*Correspondence: Chih-Chia Su (cxs670@case.edu), Carol V Robinson (carol.robinson@chem.ox.ac.uk), and Jani R Bolla (jani.bolla@biology.ox.ac.uk)*

Running title: Structural basis for lipopolysaccharide transport and lipid selectivity in *Pseudomonas aeruginosa*

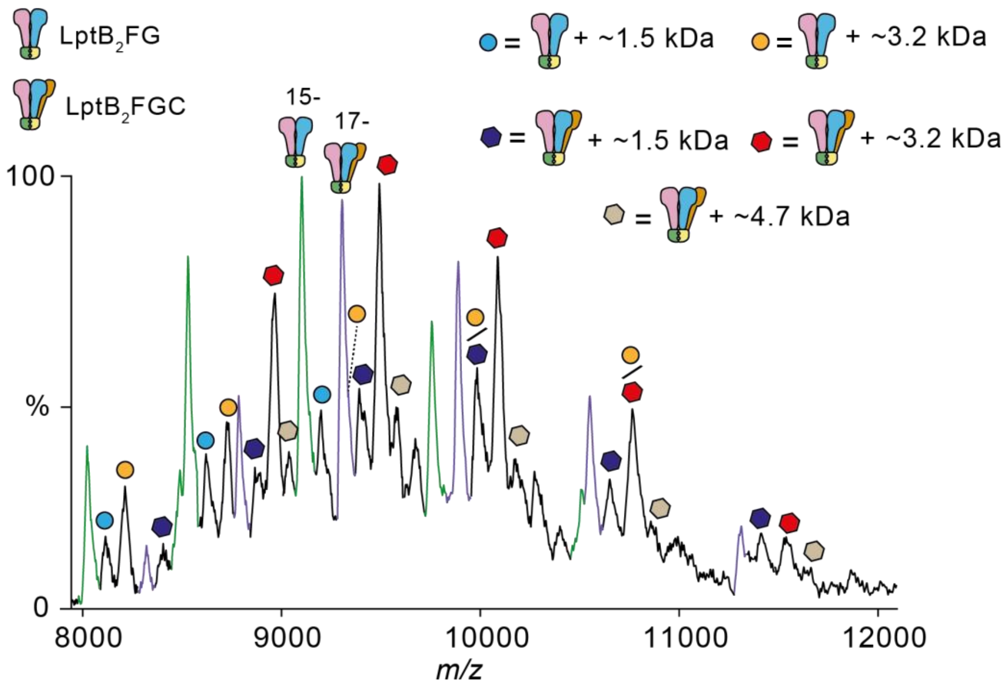

Figure S1. Native mass spectrum of LptB$_2$FG(C) expressed in *E. coli* BL21(DE3) and purified following delipidation protocol. Both LptB$_2$FG and LptB$_2$FGC are present in solution, as well as adducts ranging from 1.5 to 4.7 kDa, corresponding to a mixture of PLs and LPS co-purified with the protein complex.

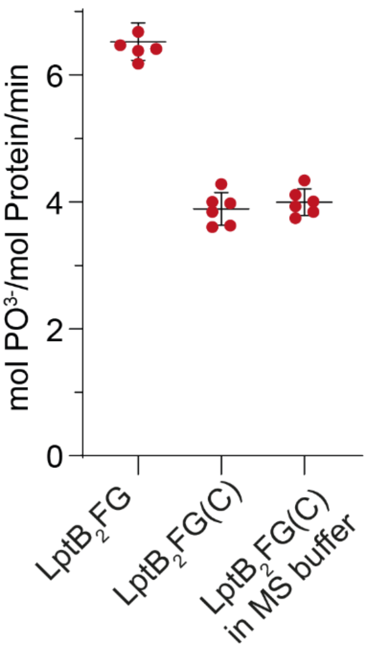

Figure S2. ATPase activities of $LptB_2FG$, $LptB_2FG$/$LptB_2FGC$ mixture and $LptB_2FG$/$LptB_2FGC$ mixture in MS buffer. Bar chart showing the ATPase activities LptB2FG, LptB2FG/LptB2FGC mixture (both in purification buffer), and LptB2FG/LptB2FGC mixture in MS buffer. Purification buffer consists of 150 mM NaCl, 20 mM Tris-HCl (pH 8.0), 10% glycerol and 0.03% DDM. MS buffer consists of 200 mM ammonium acetate (pH 8.0) and 0.04% [G1]-OGD. Activities are plotted as mean ± standard deviation (SD) of six biological replicates (n = 6).

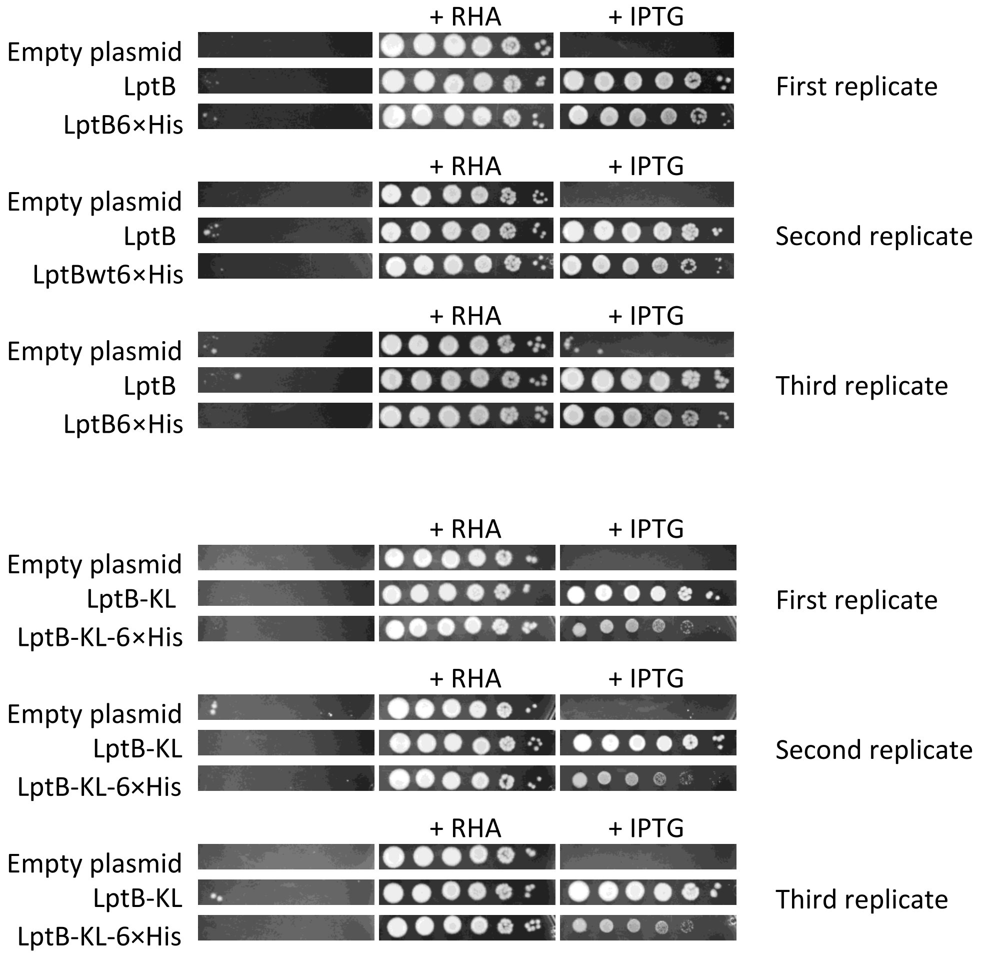

Figure S3: Histidine tagged LptB is functional *in vivo*. The lptB conditional mutant (PAO1 rhaSR-PrhaBAD-*lptB* Δ*lptB*) carrying plasmids express LptB of *P. aeruginosa* PAO1 with or without the 6×His tag at the C-terminus (LptB6×His and LptB, respectively) or the LptB variant encoded by the plasmid pQLinkN-yh-LptB-KL-6×His-FGC (construct used in this study) with or without the 6×His tag at the C-terminus (LptB-KL-6×His and LptB-KL, respectively) was plated onto Mueller-Hinton agar plates supplemented or not with 0.01% rhamnose (to induce the chromosomal copy of *lptB*) or 0.5 mM IPTG (to induce the *lptB* allele cloned into the plasmid pME6032). The conditional mutant carrying the empty plasmid pME6032 was used as the negative control. All three biological replicates are shown in the figure.

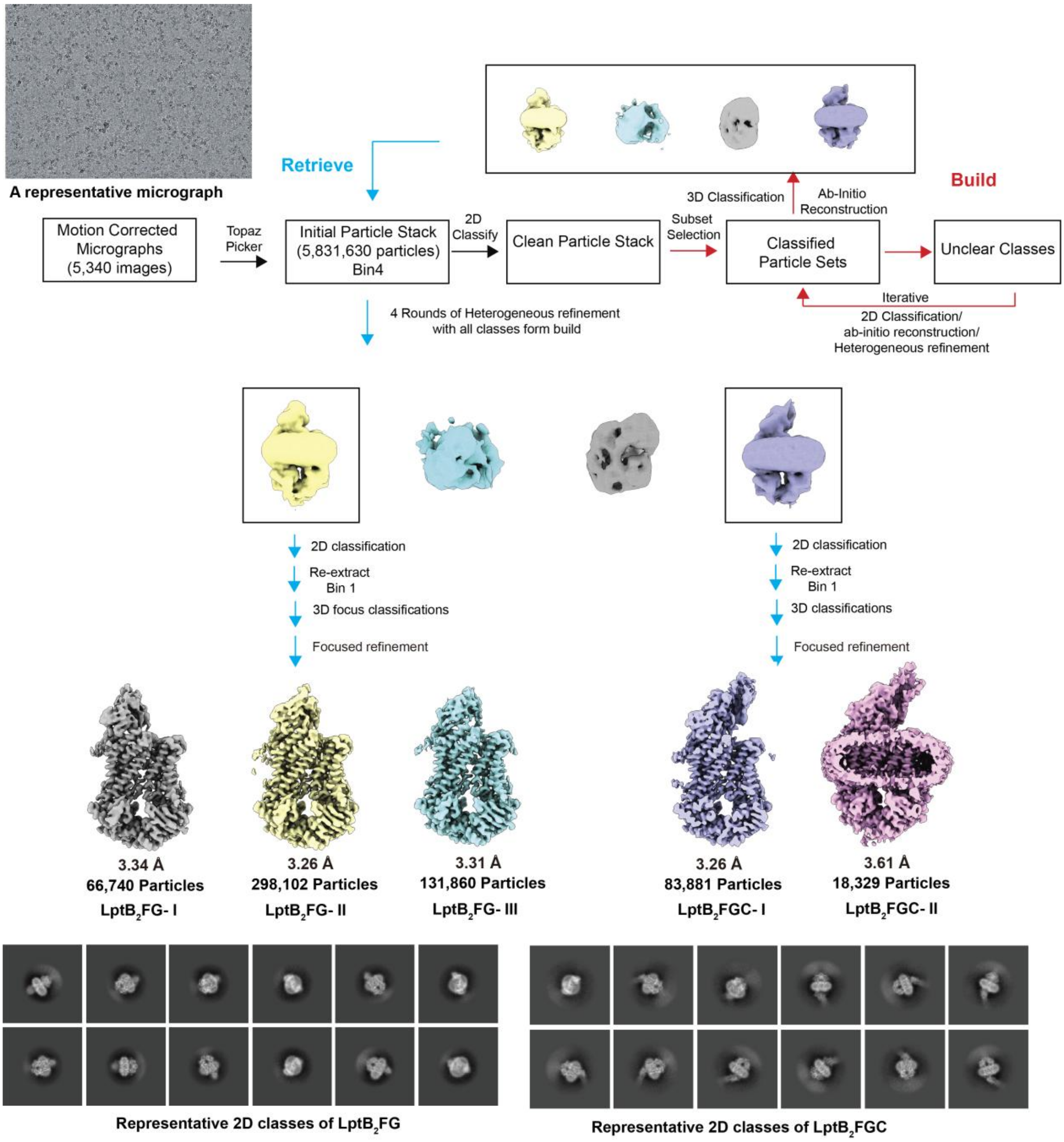

Figure S4. Cryo-EM data collection and processing LptB2FG. BaR cryo-EM data processing workflow for the LptB$_2$FG(C) complex reconstituted in lipid nanodiscs. The figure includes a representative micrograph showing particle distribution, major 3D classes obtained during refinement and classification and selected 2D class averages highlighting distinct structural features.

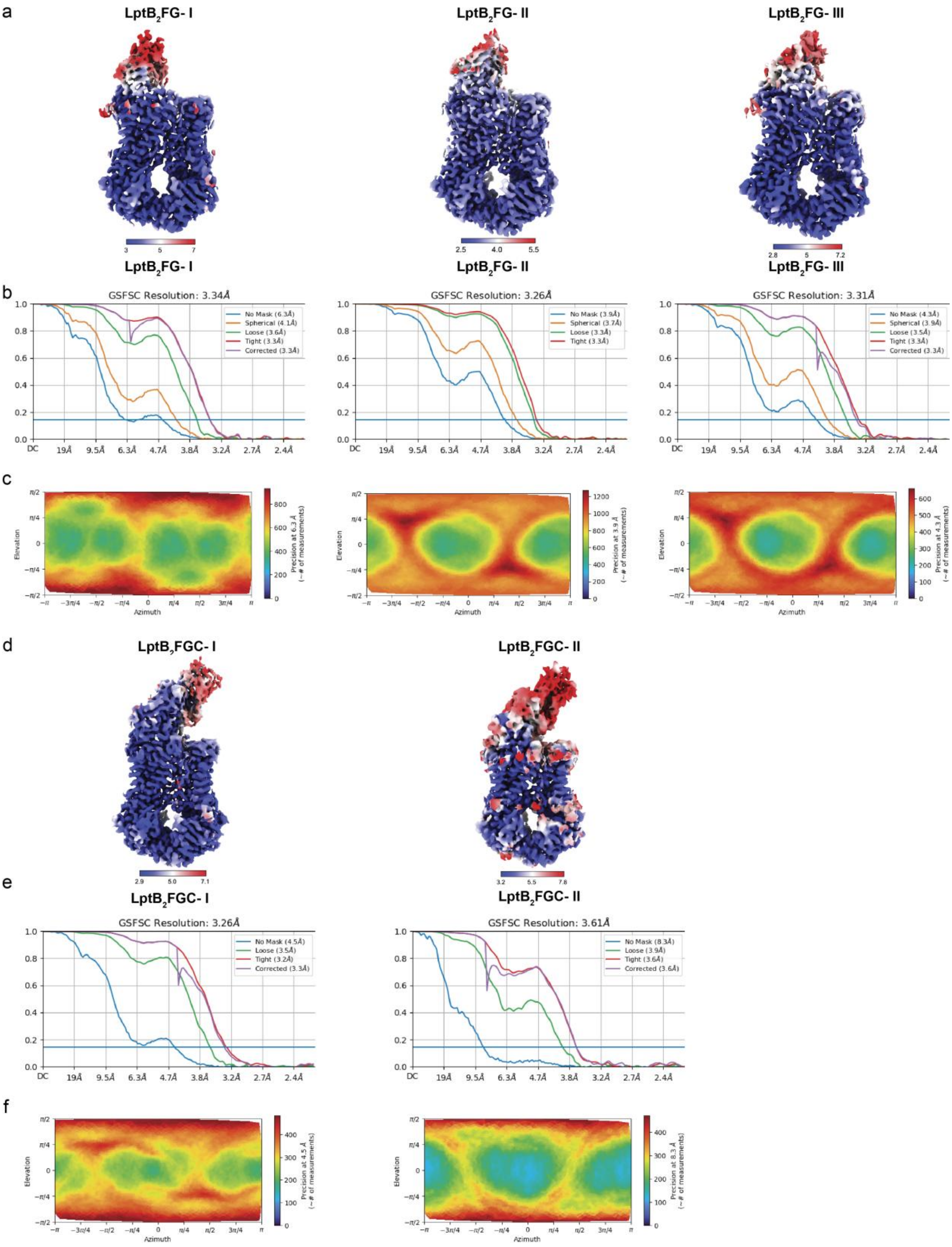

Figure S5. Cryo-EM analysis of the LptB$_2$FG(C) transporter. (a) Cryo-EM maps of LptB$_2$FG colored according to local resolution. (b) Gold standard Fourier shell correlation (GSFSC) curves of the final LptB$_2$FG reconstruction. (c) Angular distribution of particle projections used for the LptB$_2$FG reconstruction. (d) Cryo-EM maps of LptB$_2$FGC colored according to local resolution. (e) Gold

standard FSC curves of the final $LptB_2FGC$ reconstruction. (f) Angular distribution of particle projections used for the $LptB_2FGC$ reconstruction.

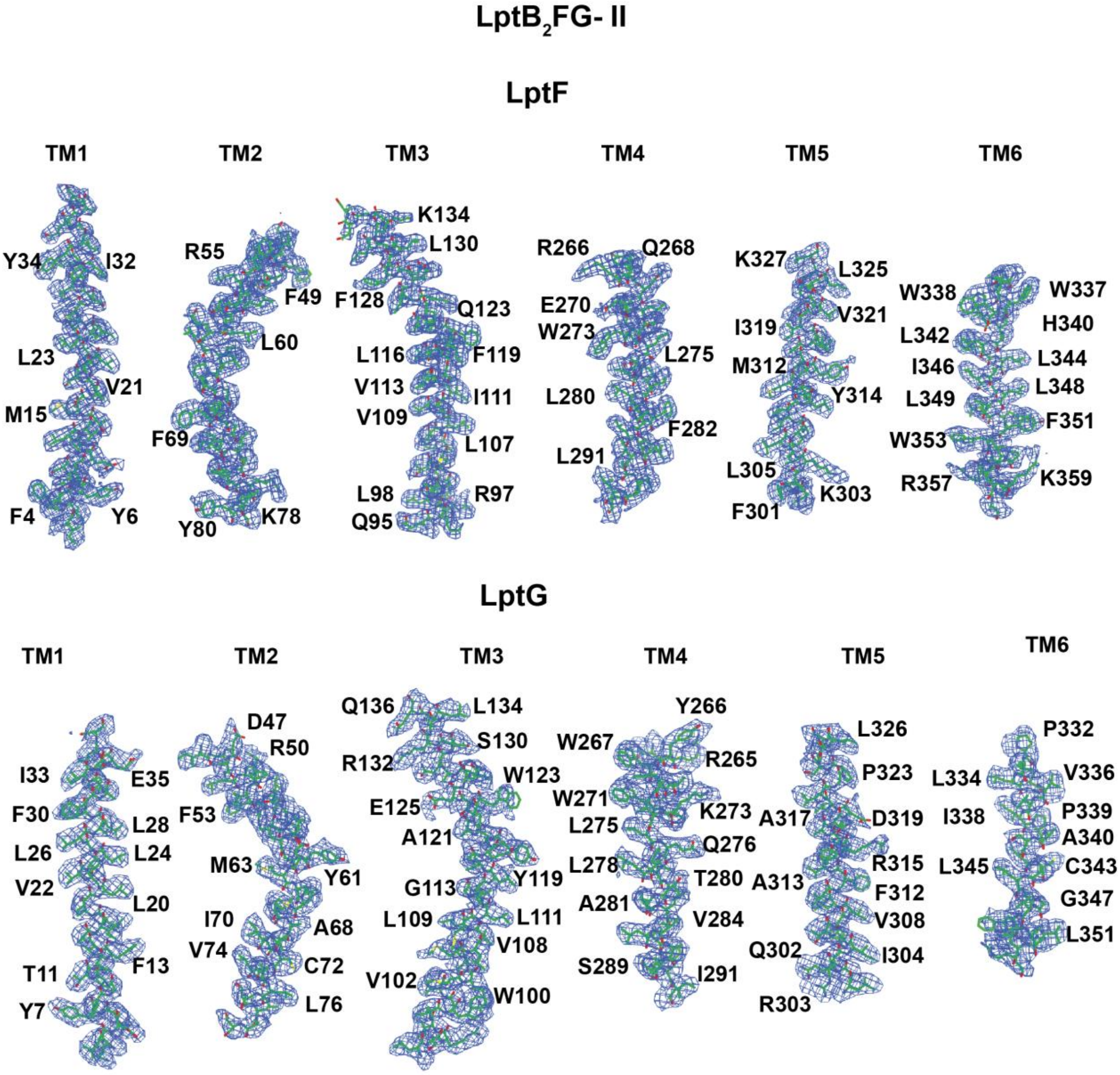

Figure S6. Cryo-EM density maps of $LptB_2FG$-II. This figure includes the structural elements of the transmembrane region of the $LptB_2FG$-II . The EM densities are in blue meshes, whereas the side chains of TMs 1-6 of LptF and LptG are in green sticks.

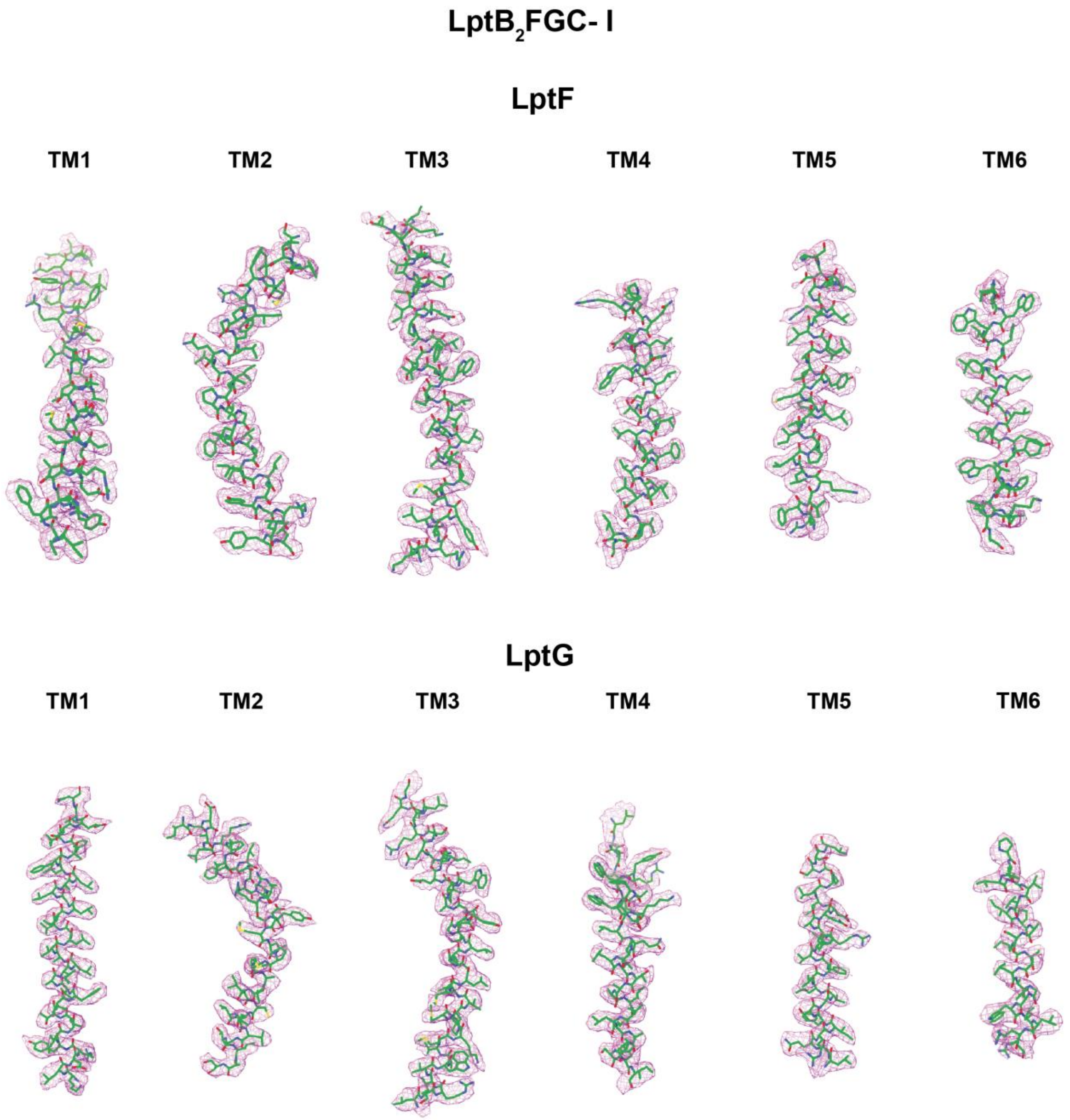

Figure S7. Cryo-EM density maps of $LptB_2FGC$-I. This figure includes the structural elements of the transmembrane region of the $LptB_2FG$-I. The EM densities are in magenta meshes, whereas the side chains of TMs 1-6 of LptF and LptG are in green sticks. The orientations of the helices are similar to those shown in Figure S6.

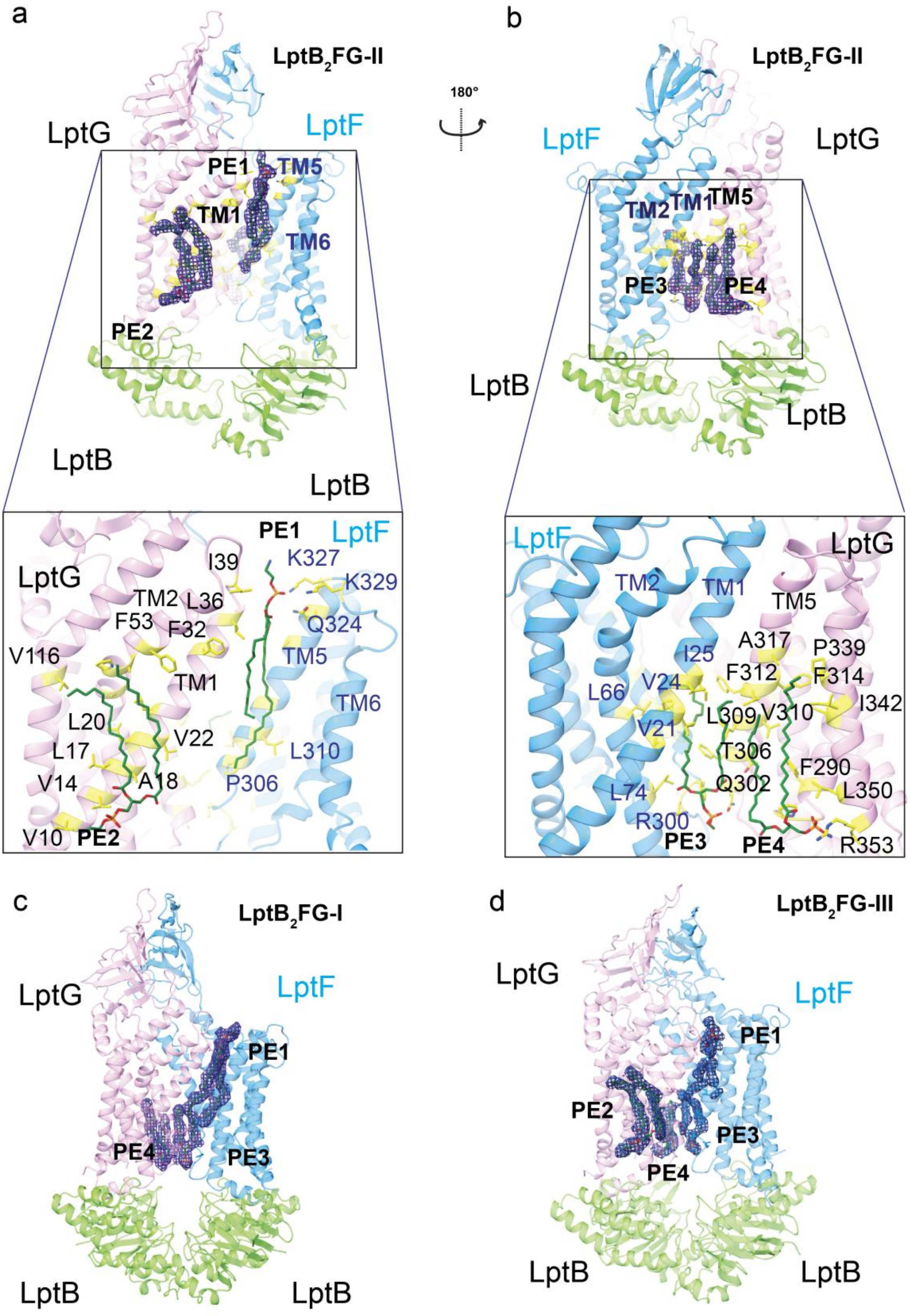

Figure S8. Three different structures of $LptB_2FG$ with PEs at the transmembrane region. (a) PE1 and PE2 are located outside of interface 1, with PE1 at the level of the outer leaflet of the cytoplasmic membrane and PE2 in the inner leaflet (b). PE3 and PE4 are situated in the inner leaflet of IM and near Interface 2. (c) PE1, PE3, and PE4 are identified in LptB2FG-I. (d) All four lipids are observed in $LptB_2FG$-III.

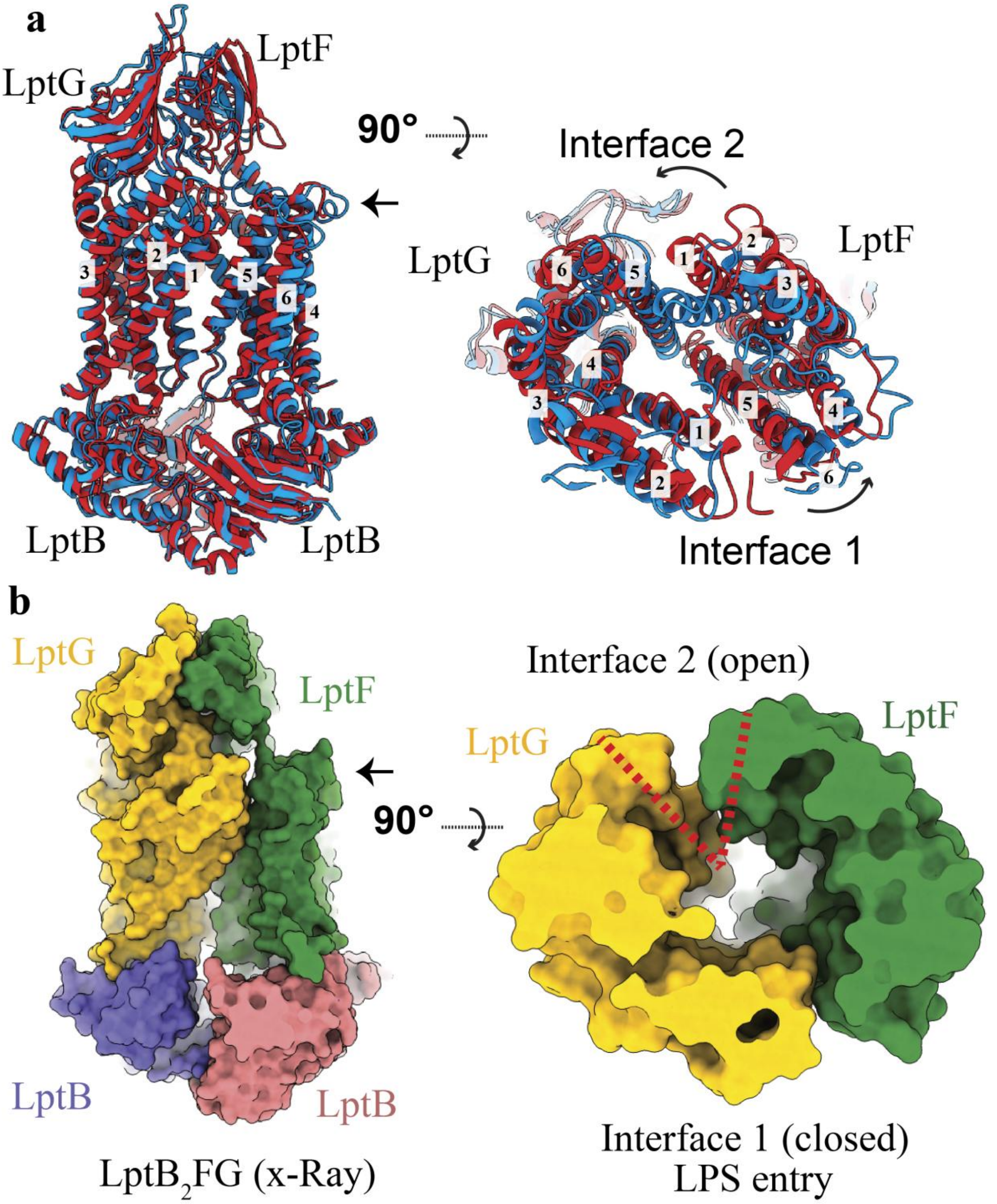

Figure S9. Structure comparisons of cryo-EM and X-ray structure of LptB$_2$FG. (a) Superimposition of X-ray (red) and cryo-EM (blue) structure of LptB$_2$FG-I. (b) Surface representation of the *P. aeruginosa* LptB$_2$FG X-ray structure. The left panel shows a membrane plane view; the black arrow indicates the approximate level of the membrane periplasmic interface at which the cross-section (right panel) was taken.

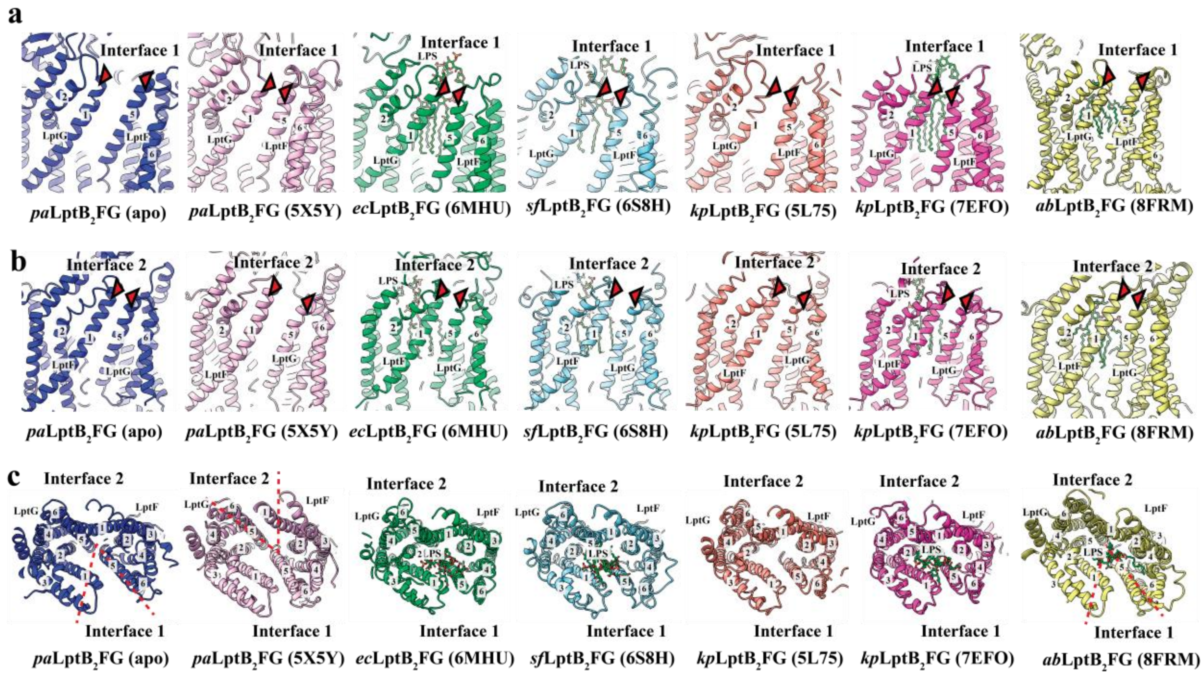

Figure S10. **Comparison of $LptB_2FG$ structures from different bacteria.** (a) Side view showing the structures of $LptB_2FG$ at interface 1. (b) Side view showing the structures of $LptB_2FG$ at interface 2. (c) Top view of $LptB_2FG$ structures highlighting the opening and closing of interface 1 and interface 2.

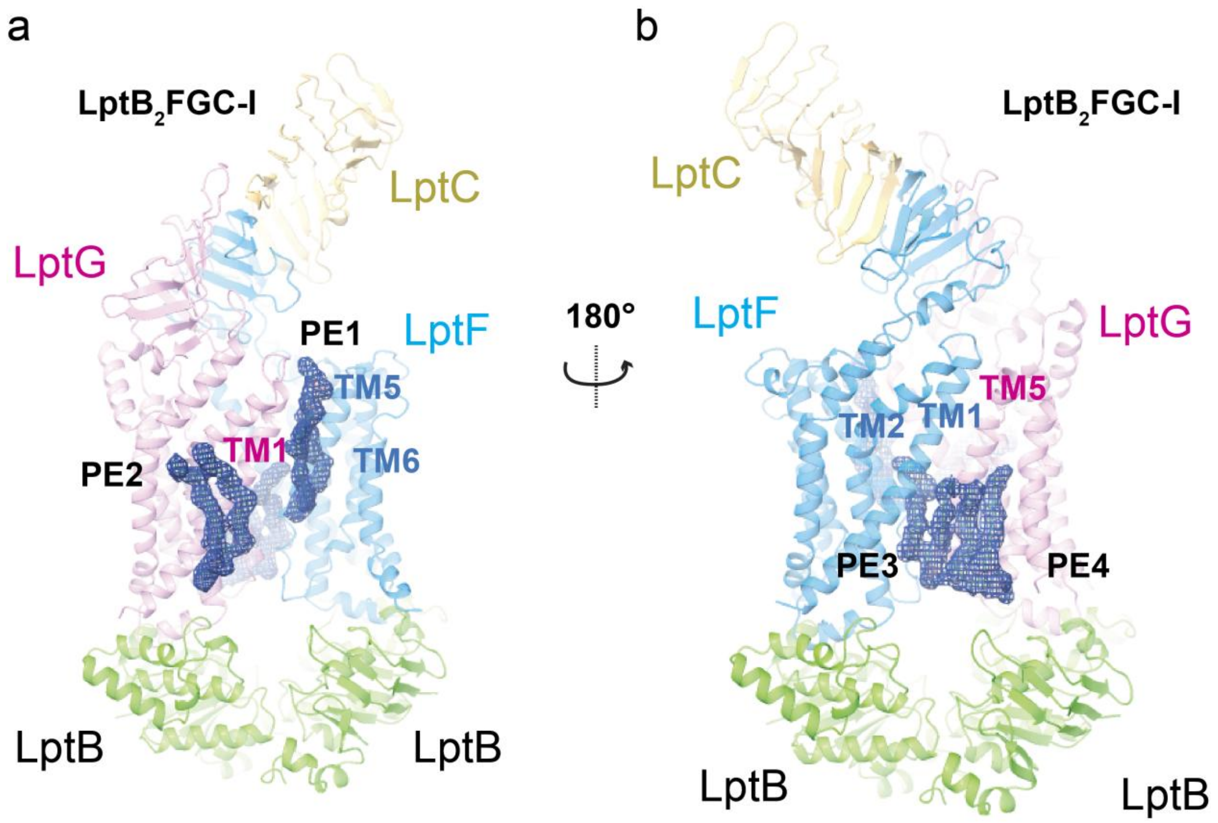

Figure S11. Structure of $LptB_2FGC$ with four PEs at the transmembrane region. (a) PE1 and PE2 are located outside of interface 1, with PE1 at the level of the outer leaflet of the IM and PE2 in the inner leaflet (b). PE3 and PE4 are situated in the inner leaflet of IM and near Interface 2.

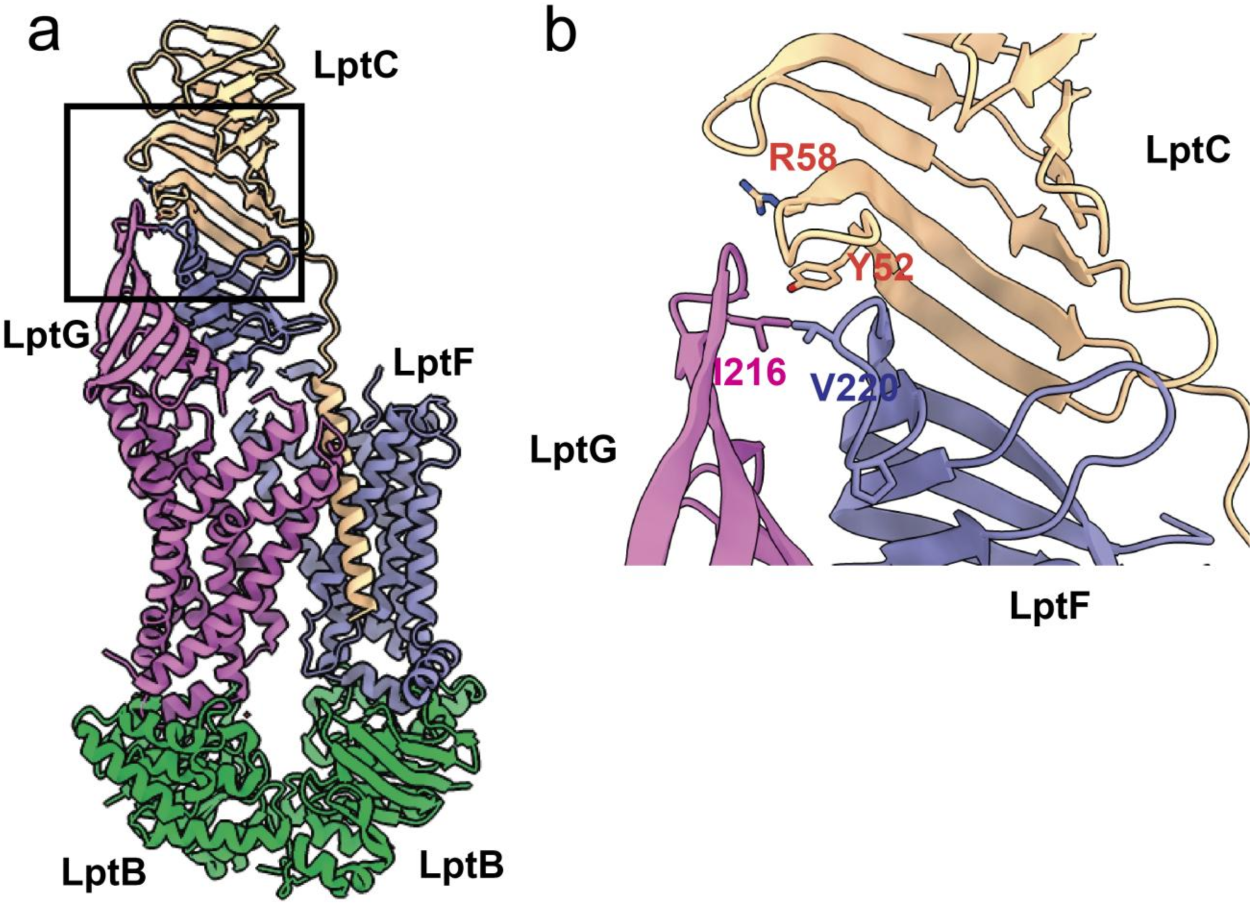

Figure S12. Interface of *V. cholerae* LptB$_2$FGC highlighting key contacts between LptC, LptF, and LptG. a) Overall structure of the *V. cholerae* LptB$_2$FGC complex showing the arrangement of LptC, LptF, and LptG, and highlighting the primary interaction region between LptC and LptF. (b) Close-up view of the LptG–LptC interface, showing residues within 3.5 Å that contribute to contacts between their β-jellyroll domains. This indicates that LptG makes only limited interactions with LptC compared to LptF.

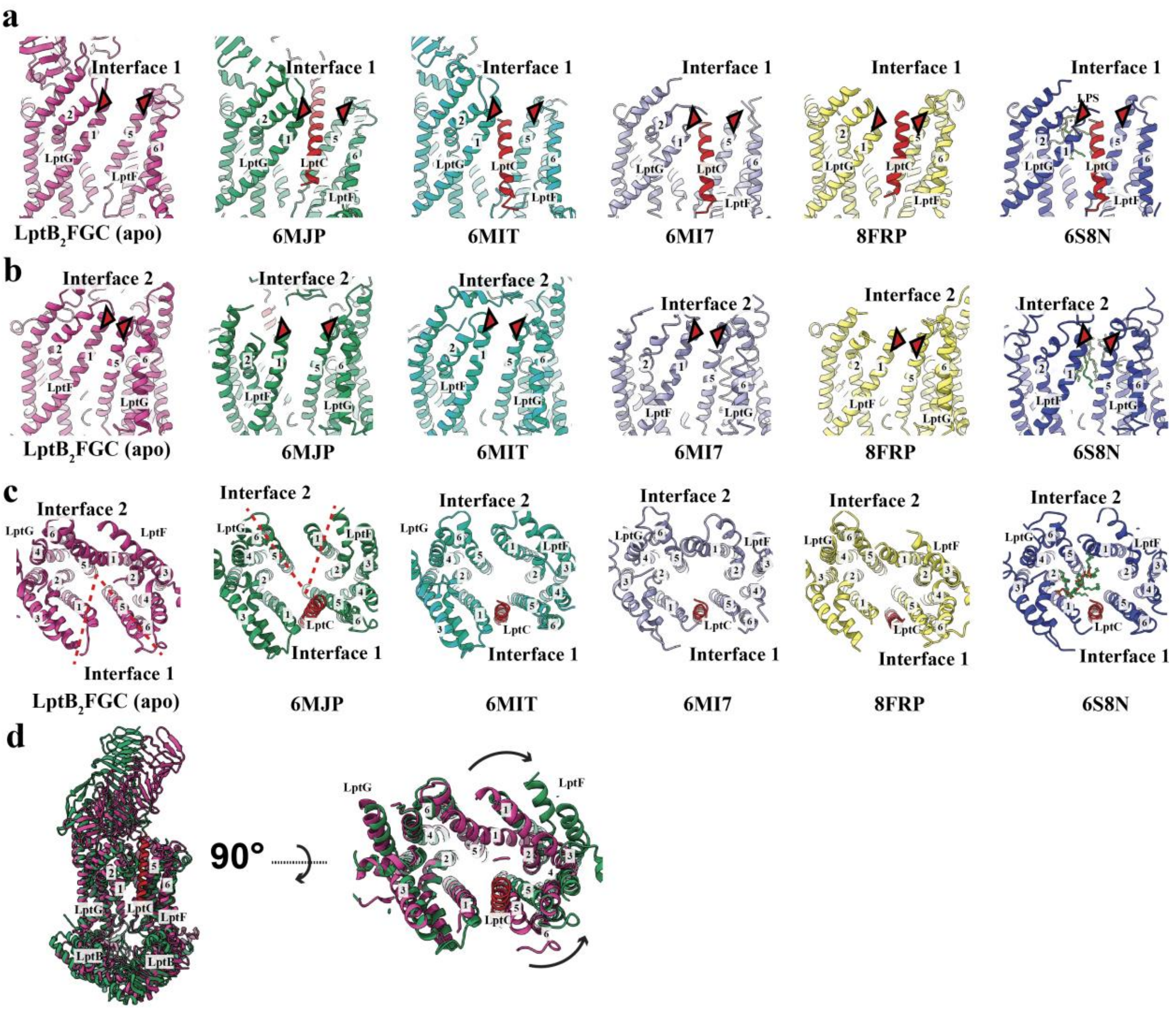

Figure S13. **Comparison of LptB$_2$FGC structures from different bacteria.** (a) Side view showing the structures of LptB$_2$FGC at interface 1. (b) Side view showing the structures of LptB$_2$FGC at interface 2. (c) Top view of LptB$_2$FGC structures highlighting the open and closed interfaces. (d) A comparison of our cryo-EM structure of LptB$_2$FGC-I (magenta) with the previously reported *V. cholerae* LptB$_2$FGC (6MJP) (green), reveals a significant conformational change.

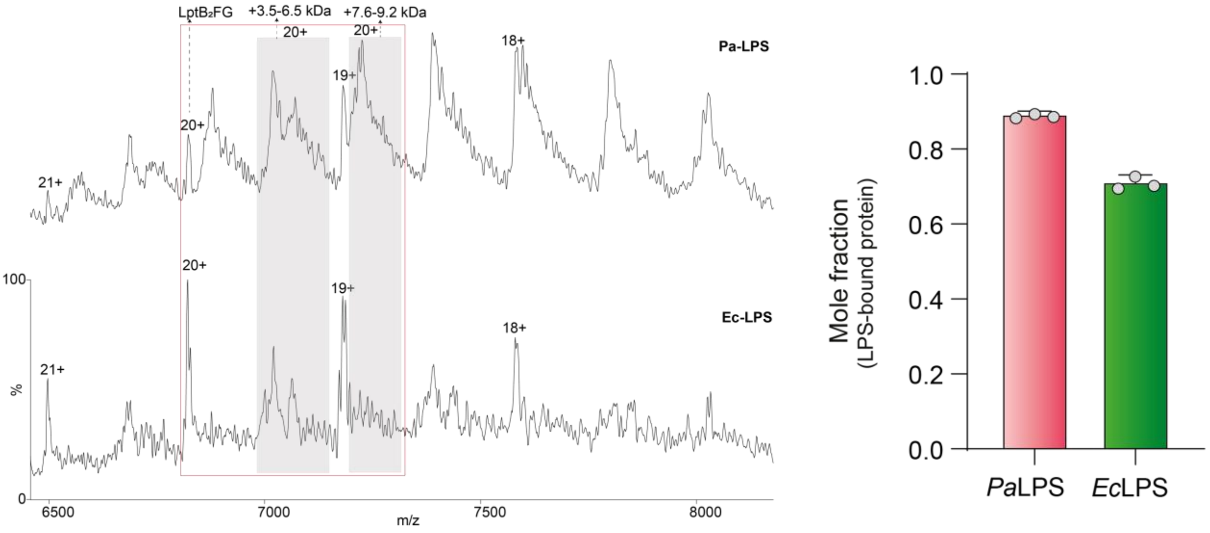

Figure S14. Pa- LptB$_2$FG binds its native LPS with higher affinity than *E. coli* LPS. Native mass spectra of Pa-LptB2FG in the presence of *P. aeruginosa* LPS (top) and *E. coli* LPS (bottom). 0.5 µL of LPS at 5 mg/ml was added to 10 µL of purified LptB2FG at 3 µM concentration. Since LPS is an heterogenous mixture, a number of adduct peaks are observed in both cases. The bar chart on the right shows the relative quantification, suggesting that LPS binding is much higher in the case of Pa-LPS compared to Ec-LPS. Data are expressed as mean ± standard deviation (SD) of three biological replicates (n = 3). We explicitly caution that these values are semi-quantitative because of analyte heterogeneity.

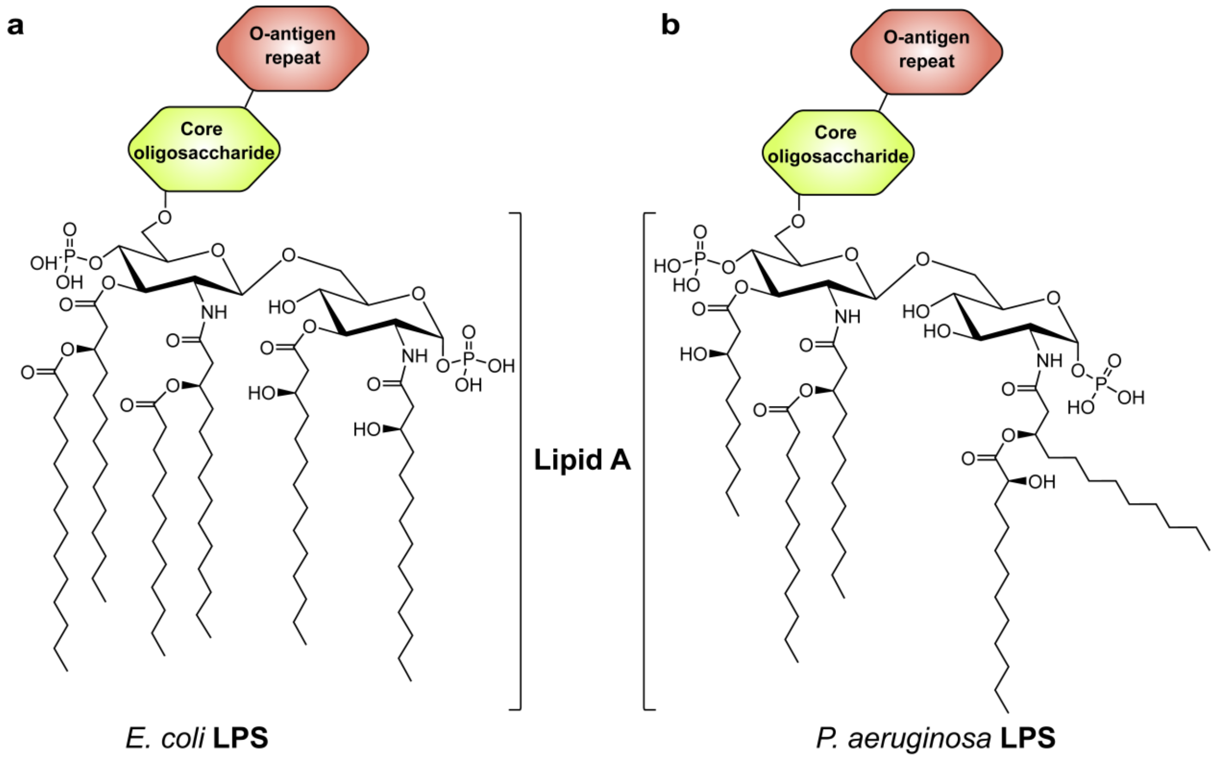

Figure S15: Structures of LPS from *E. coli* and *P. aeruginosa*. The structures are differed by the number of acyl chains in the lipid A core, in addition to subtle differences in oligosaccharides.

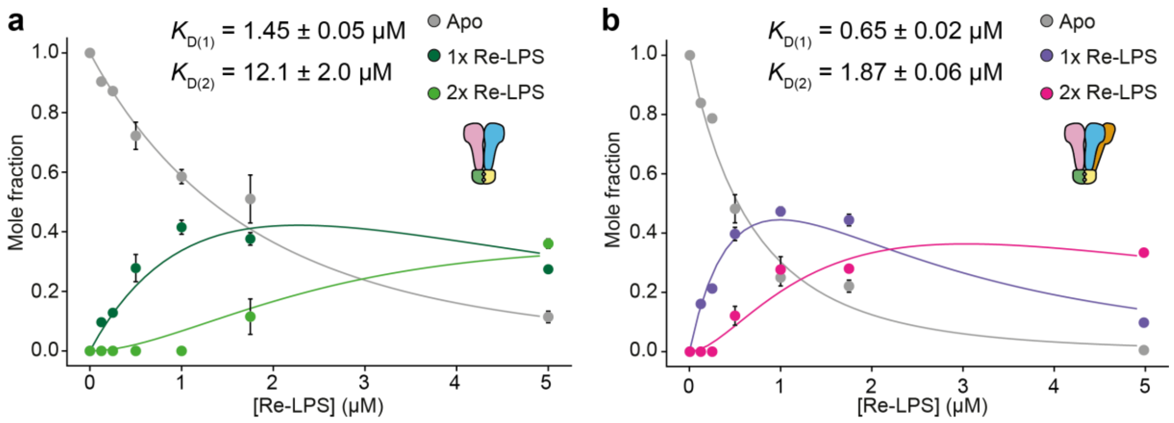

Figure S16. Quantification of Re-LPS binding to $LptB_2FG$ and $LptB_2FGC$. Plot of mole fraction for each Re-LPS-bound state of LptB2FG (a) and LptB2FGC (b) as a function of total [Re-LPS]. Solid lines show the fit to an equilibrium binding model to determine the apparent KDs for the first and second lipid binding events. Data are expressed as mean ± standard deviation (SD) of three biological replicates (n = 3).

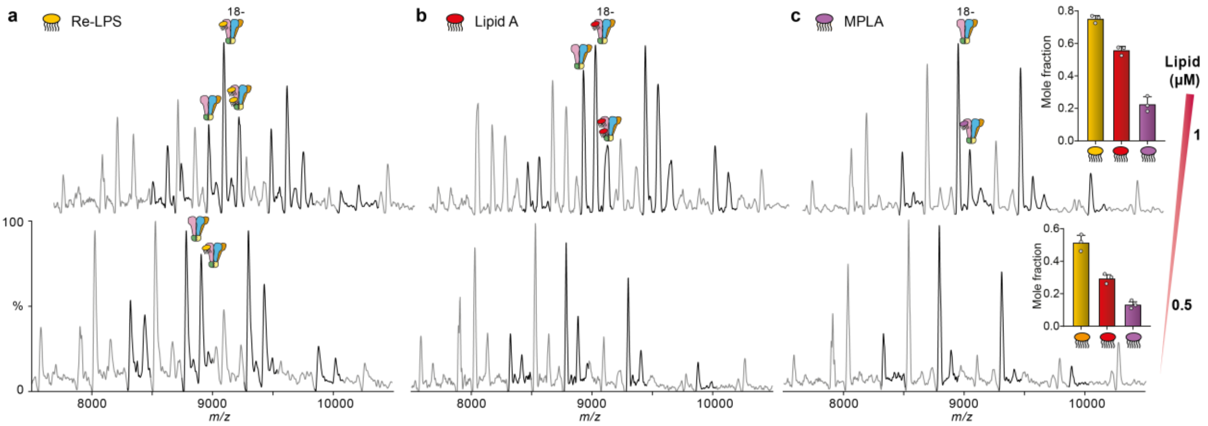

Figure S17. **Native MS analysis of the interaction between LptB$_2$FG/LptB$_2$FGC and Re-LPS, Lipid A and MPLA.** Native mass spectra of LptB2FG/LptB2FGC (total protein concentration of 3 µM) following incubation with Re-LPS (a), Lipid A (b) and MPLA (c) at 0.5 and 1 µM. Charge state series indicating lipid binding were observed, and the total amount of lipid-bound protein complex was calculated. The bar charts show the quantification of lipid-bound species and suggest that the amount of lipid binding decreases as the number of phosphate groups decreases (inset). Data are expressed as mean ± standard deviation (SD) of three biological replicates (n = 3).

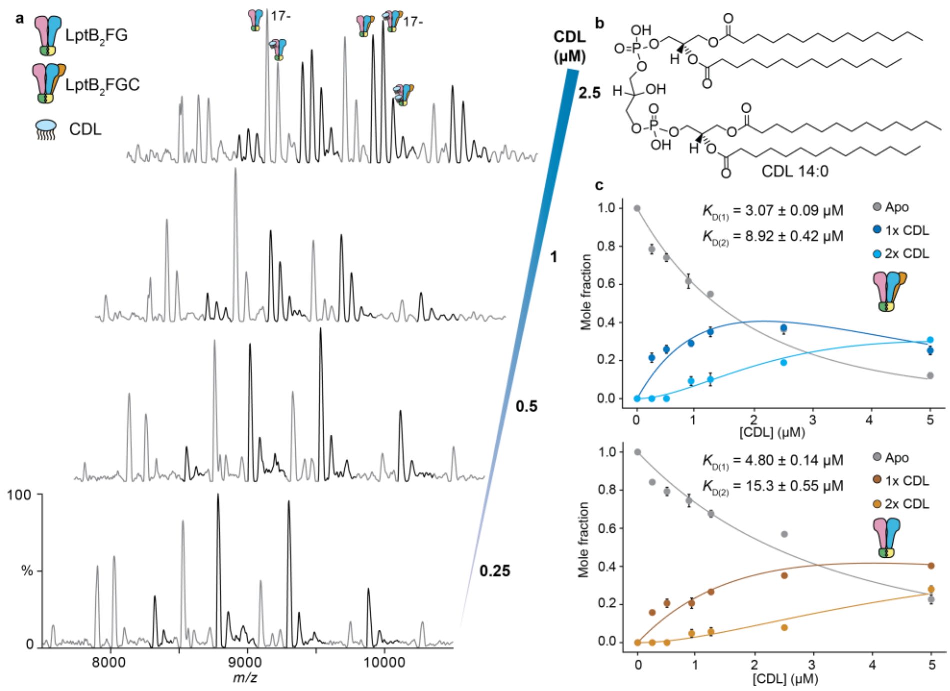

Figure S18. Quantification of CDL binding to $LptB_2FG$ and $LptB_2FGC$. (a) Native mass spectra of LptB2FG/LptB2FGC (total protein concentration of 3 μM) following incubation with CDL at increasing concentrations. Charge state series indicating lipid binding were observed and the total amount of CDL bound protein complex was calculated. (b) Molecular structure of CDL. (c) Plot of mole fraction for each CDL-bound state of LptB2FG (lower panel) and LptB2FGC (upper panel) as a function of total [CDL]. Solid lines show the fit to an equilibrium binding model to determine the apparent KDs for the first and second lipid binding events. Data are expressed as mean ± standard deviation (SD) of three biological replicates (n = 3).

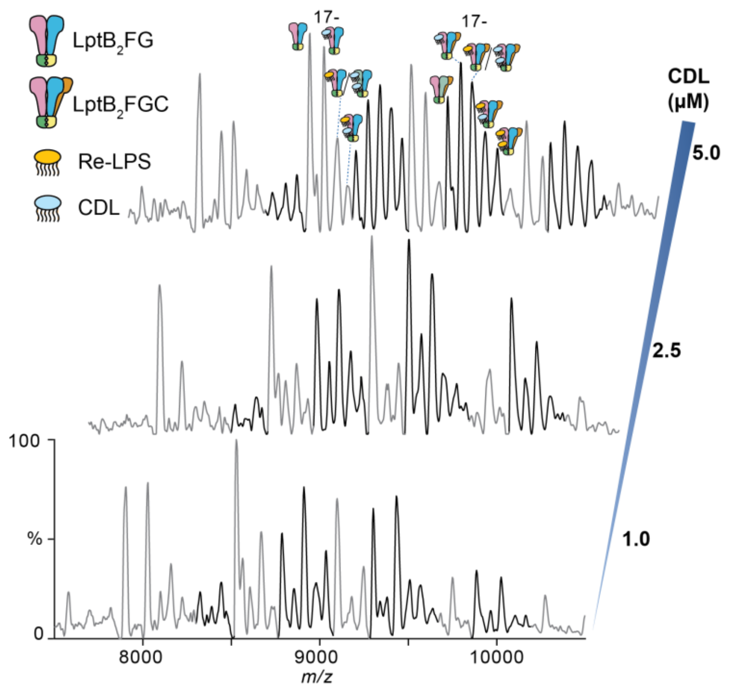

Figure S19. Influence of CDL on the interaction between Re-LPS and LptB$_2$FG/LptB$_2$FGC. Native mass spectra of LptB$_2$FG/LptB$_2$FGC (total protein concentration of 3 μM) in the presence of Re-LPS (fixed concentration of 1 μM) and increasing concentrations of CDL.

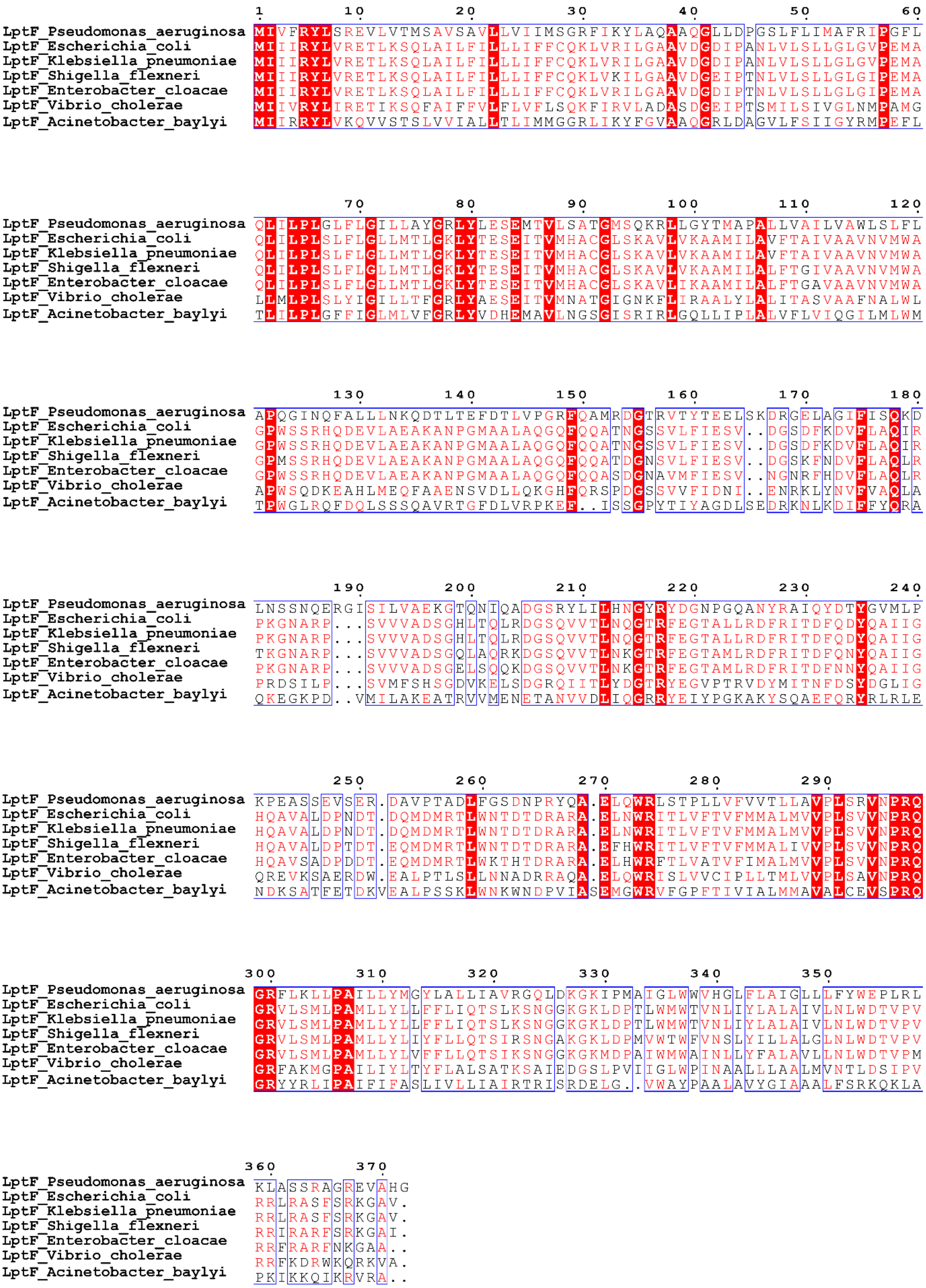

Figure S20. Sequence alignment of *P. aeruginosa* LptF with its orthologues from *E. coli*, *K. pneumoniae*, *S. flexneri*, *E. cloacae*, *V. cholerae*, and *A. baylyi.* Red colour indicates conserved residues, highly similar residues are shown in bold. Alignments generated with NPS@ClustalW and ESPript 3.0.

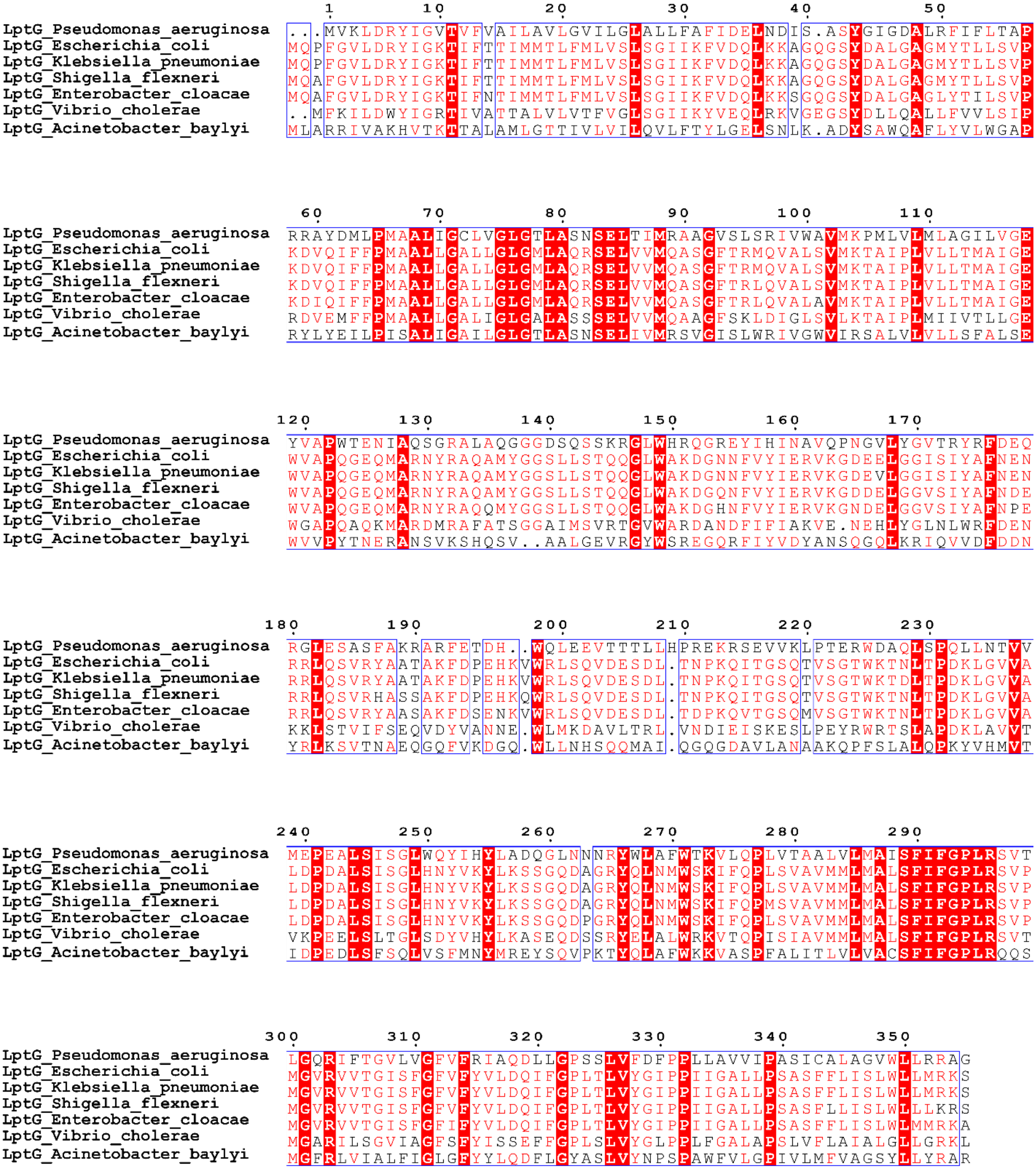

Figure S21. Sequence alignment of *P. aeruginosa* LptG with its orthologues from *E. coli*, *K. pneumoniae*, *S. flexneri*, *E. cloacae*, *V. cholerae*, and *A. baylyi*. Red colour indicates conserved residues, highly similar residues are shown in bold. Alignments generated with NPS@ClustalW and ESPript 3.0.

Table S1. Cryo-EM data collection, processing, and refinement statistics for $LptB_2FG$ and $LptB_2FGC$.

**Apo**<br>**Data set**	**B_2FG-I**	**B_2FG-II**	**B_2FG-III**	**B_2FGC-I**	**B_2FGC-II**
**Data collection and processing**					
Magnification			81,000		
Voltage (kV)			300		
Electron Microscope			Krios-GIF-K3		
Defocus range (μm)			-1.0 to -2.5		
Total exposure time (s)			2		
Pixel size (Å)			1.08		
Total dose (e^-/ $Å^2$)			50.0		
Number of frames			17.3		
Does rate (e^-/ phys. Pixel/s)					
No. of initial micrographs			7,853		
No. of initial particles			16,247,216		
No. of final particles	66,740	298,102	131,860	83,881	18,329
Symmetry	C1	C1	C1	C1	C1
GSFSC Resolution (Å)	3.34	3.26	3.31	3.26	3.61
FSC threshold (0.143)					
Map resolution range (Å)	3.02-30	2.71-30	2.82-30	2.93-30	3.20-30
**Refinement**					
Model resolution cut-off (Å)	3.34	3.26	3.31	3.26	3.61
Model composition					
No. of Protein residues	1194	1198	1186	1330	1324
No. of ligands	3	4	4	4	1
**RMSD**[a]					
Bond lengths (Å)	0.004	0.003	0.003	0.002	0.003
Bond angles (°)	0.572	0.523	0.560	0.411	0.596
**Validation**					
MolProbity score	1.83	1.59	1.75	2.27	1.73
Clash score	7.96	6.40	8.19	27.91	9.58
Rotamer outliers (%)	0.21	0	0	0	0
**Ramachandran plot (%)**					
Favored (%)	94.10	96.39	95.66	95.08	96.58
Allowed (%)	5.90	3.61	4.34	4.92	3.42
Disallowed (%)	0	0	0	0	0
CC mask	0.85	0.86	0.84	0.85	0.83
CC box	0.70	0.76	0.76	0.70	0.71
CC vol	0.82	0.81	0.81	0.82	0.82
EMD-	47084	47085	47086	47088	47089
PDB ID	9DOH	9DOK	9DOO	9DOQ	9DOR

[a], root mean square deviation;

Table S2. Summary of interactions between the glucosamine phosphate groups and LptF/LptG residues in reported $LptB_2FG(C)$–LPS structures

	position 1		position 4	
	LptG	LptF	LptG	LptF
A. Baylyi $LptB_2FG$ (8FRM)		K33		R30, R55
K. pneumoniae $LptB_2FG$ (7EFO)	R133			E58
S. flexneri $LptB_2FG$ (6S8H)	R133			
S. flexneri $LptB_2FGC$ (6S8N)	K62			
E. Coli $LptB_2FG$ (6MHU)	K34, R133			K30

Uncropped SDS-PAGE gel for image shown in Figure 1b

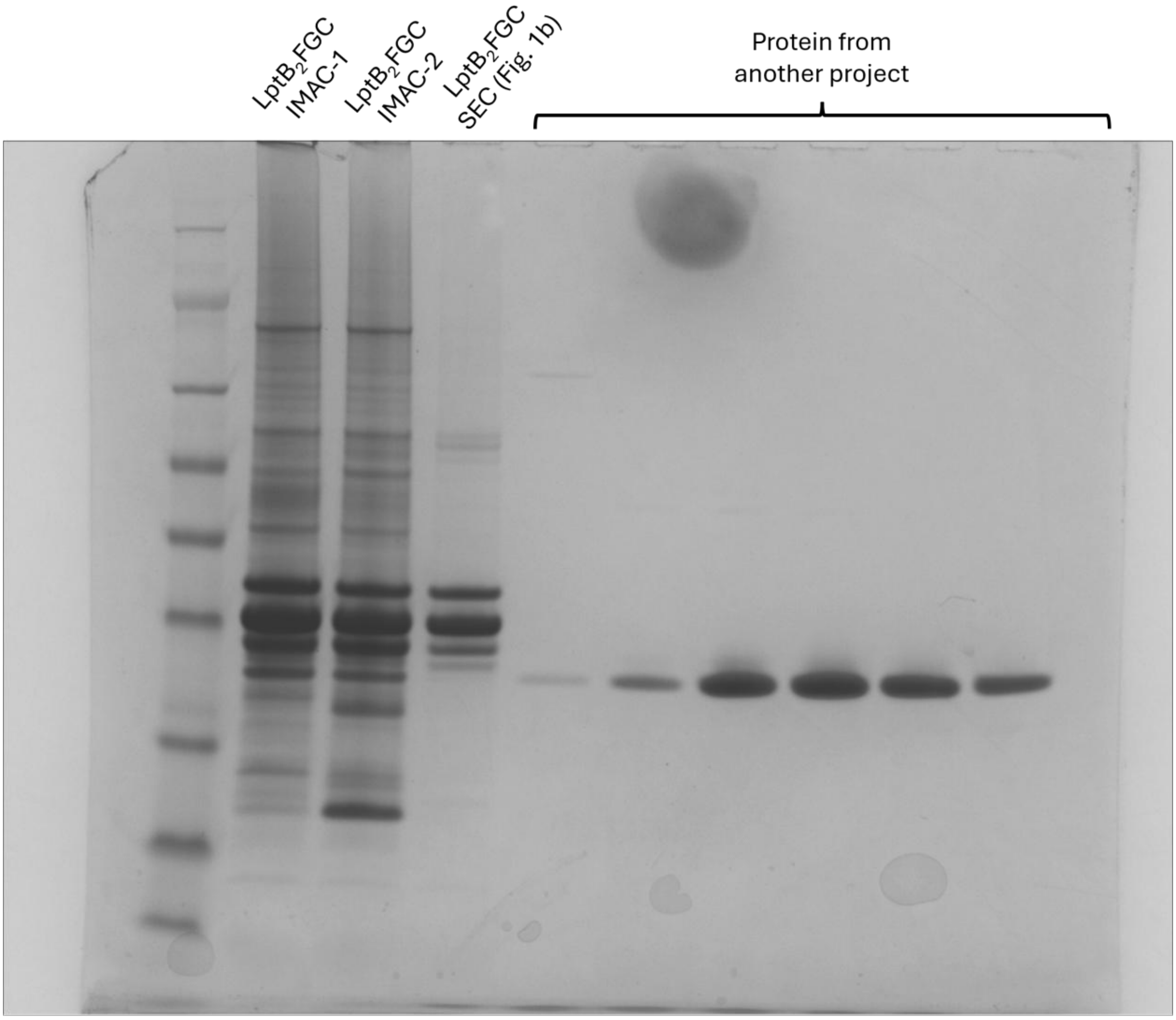

Supplement: Supplementary file 1 — Supplementary Information [file 41467_2025_66182_MOESM1_ESM.pdf]
